# Reliability of clinical tests to evaluate nerve function and mechanosensitivity of the upper limb peripheral nervous system

**DOI:** 10.1186/1471-2474-10-11

**Published:** 2009-01-21

**Authors:** Annina B Schmid, Florian Brunner, Hannu Luomajoki, Ulrike Held, Lucas M Bachmann, Sabine Künzer, Michel W Coppieters

**Affiliations:** 1 Uniklinik Balgrist, Department of Physiotherapy, Forchstrasse 340, 8008 Zurich, Switzerland; 2 Uniklinik Balgrist, Department of Rheumatology, Forchstrasse 340, 8008 Zurich, Switzerland; 3 Zurich University of Applied Sciences, School of Health Professions, Institute of Physiotherapy, Technikumstrasse 71, 8401 Winterthur, Switzerland; 4 Horten Center for Patient-Oriented Research, Bolleystrasse 40, Postfach Nord, University of Zurich, Zurich, Switzerland; 5 School of Health and Rehabilitation Sciences, The University of Queensland, Brisbane, Queensland, Australia

## Abstract

**Background:**

Clinical tests to assess peripheral nerve disorders can be classified into two categories: tests for afferent/efferent nerve function such as nerve conduction (bedside neurological examination) and tests for increased mechanosensitivity (e.g. upper limb neurodynamic tests (ULNTs) and nerve palpation). Reliability reports of nerve palpation and the interpretation of neurodynamic tests are scarce. This study therefore investigated the intertester reliability of nerve palpation and ULNTs. ULNTs were interpreted based on symptom reproduction and structural differentiation. To put the reliability of these tests in perspective, a comparison with the reliability of clinical tests for nerve function was made.

**Methods:**

Two experienced clinicians examined 31 patients with unilateral arm and/or neck pain. The examination included clinical tests for nerve function (sensory testing, reflexes and manual muscle testing (MMT)) and mechanosensitivity (ULNTs and palpation of the median, radial and ulnar nerve). Kappa statistics were calculated to evaluate intertester reliability. A meta-analysis determined an overall kappa for the domains with multiple kappa values (MMT, ULNT, palpation). We then compared the difference in reliability between the tests of mechanosensitivity and nerve function using a one-sample t-test.

**Results:**

We observed moderate to substantial reliability for the tests for afferent/efferent nerve function (sensory testing: kappa = 0.53; MMT: kappa = 0.68; no kappa was calculated for reflexes due to a lack of variation). Tests to investigate mechanosensitivity demonstrated moderate reliability (ULNT: kappa = 0.45; palpation: kappa = 0.59). When compared statistically, there was no difference in reliability for tests for nerve function and mechanosensitivity (p = 0.06).

**Conclusion:**

This study demonstrates that clinical tests which evaluate increased nerve mechanosensitivity and afferent/efferent nerve function have comparable moderate to substantial reliability. To further investigate the clinometric properties of these tests, more studies are needed to evaluate their validity.

## Background

Bedside neurological examination (sensory testing, reflexes and MMT) is used to evaluate lesions of the peripheral nervous system [1]. This assessment screens for nerve injuries characterised by changes in afferent or efferent nerve function such as changes in nerve conduction [2]. Beside these nerve lesions, various other nerve disorders do not necessarily lead to altered afferent or efferent function [3,4]. An inflamed nerve, for example, can be highly sensitive to mechanical stimuli [5], such as compression and stretch, but conduction velocity through the inflamed region can be near normal [6]. Therefore, when relying solely on traditional bedside neurological examination, nerve lesions characterised by increased sensitivity to mechanical stimuli may be overlooked.

With body movements the nervous system slides relative to its surrounding structures and is subject to substantial compression and stretch [7-10]. Whereas a healthy nervous system can tolerate this loading, low levels of stretch and compression are sufficient to generate ectopic impulses from an inflamed nerve [5,6]. This increased mechanosensitivity is the key characteristic that is being evaluated in many clinical provocation tests, such as Spurling's test for cervical radiculopathy and the straight leg raising test for lumbar radiculopathy [11,12].

In accordance with the straight leg raising test, neurodynamic tests for the upper limb were designed to evaluate the mechanosensitivity of the brachial plexus and the median, radial and ulnar nerve [13,14]. A neurodynamic test is considered positive if symptoms can be reproduced and if symptoms can be altered by structural differentiation [13]. Structural differentiation uses movement at a site remote to the painful area to further load or unload the nervous system [13,15]. An example is the addition of ankle dorsiflexion to a straight leg raising test to alter radicular symptoms. The reliability of neurodynamic tests for the upper limb (ULNTs) has been explored widely [16-23]. The majority of these studies investigated whether symptom reproduction occurred at a consistent point through range. The overall view is that range of motion measurement at the point of symptom reproduction is reliable.

Although structural differentiation is an important criterion for the interpretation of neurodynamic tests [24,25], to our knowledge, there is only one reliability study that included structural differentiation as one of the criteria for a positive test. Wainner et al. [26] observed substantial intertester agreement (kappa: 0.76 to 0.83). A limitation of this study is that a change in symptoms with structural differentiation was not an essential criterion for a test to be considered positive. A test was also positive if only symptoms were reproduced or if a side difference in range of motion was observed. As such, there is no study available that investigates the reliability of the interpretation of neurodynamic tests with structural differentiation as an essential criterion.

Beside ULNTs, nerve palpation has also been proposed to investigate the mechanosensitivity of the nervous system [25,27]. To our knowledge, only one study has examined the intertester reliability of this method. Jepsen et al. [28] demonstrated moderate to substantial reliability for palpation of the nerves of the upper limb. Additional studies to test the reliability of nerve palpation are required.

The aim of this study was to evaluate the intertester reliability of nerve palpation and the reliability of the interpretation of ULNTs when symptom reproduction and structural differentiation are considered essential criteria. Although ULNTs are the equivalent of the straight leg raise for the upper limb, ULNTs are in our opinion less commonly used and not as frequently performed as the bedside neurological examination. This might be due to the fact that ULNTs are somewhat more complex to perform, which may impact on their reliability. To put the tests for mechanosensitivity in perspective, a comparison with the reliability of the clinical tests for afferent and efferent nerve function was made.

## Methods

### Participants

Thirty one patients (15 men and 16 women) were recruited from the Rheumatology and Physiotherapy Department of the Balgrist University Hospital in Zurich, Switzerland. Sample size calculation was based on identifying a moderate strength of agreement at a significance level of 0.05 and a power of 80% [29].

Patients were included if they presented with unilateral, non-acute arm and/or neck pain (≥ 1 months duration) and were between 18 and 60 years old (mean age 44 (SD ± 11.5 years)). Patients were excluded if they had underlying diseases, such as diabetes mellitus, widespread neurological disorders, upper limb/spinal surgery or significant trauma in the preceding 3 months, spinal cord or cauda equina signs, cancer or inflammatory disorders.

The patients presented with 14 different clinical diagnoses as defined by their general practitioners (Table 1). The median symptom duration was 10 months. Approval was obtained from the Ethics Committee of Balgrist University Hospital. All patients gave written consent to participate in the study.

**Table 1:** Included diagnoses

Diagnosis*	**Number of cases**
Cervical radiculopathy	2
Cervical disc herniation	2
Nonspecific neck pain	4
Nonspecific arm pain	6
Rotator cuff tear	2
Biceps and supraspinatus tendinopathy	1
Shoulder impingement	2
Persistent pain after proximal ulnar fracture	1
Neurolysis of the ulnar nerve at elbow	1
Cubital tunnel syndrome	1
Lateral epicondylalgia	2
Nonspecific paraesthesia in the hand	1
Nonspecific wrist pain	3
Carpal Tunnel Syndrome	3

*Diagnosis as referred by general practitioners, based on clinical findings, supported with imaging techniques where required.

### Examination

The examination consisted of three parts which were performed in a standardised order: (1) bedside neurological examination, (2) ULNTs for the median (two most commonly used variants), radial and ulnar nerve, and (3) palpation of the peripheral nerves.

#### Bedside neurological examination

Bedside neurological examination consisted of manual muscle tests, sensory testing and reflex testing. MMTs were performed for the myotomes C4 to T1 (C4: upper trapezius; C5: middle deltoid; C6: biceps brachii; C7: triceps brachii; C8: extensor hallucis longus; T1: palmar interossei). All MMTs were performed using the methods described by Kendall and McCreary [30]. Muscle strength was rated as normal or decreased.

Sensory testing evaluated sensitivity for light touch. It was examined from the dermatome C4 downwards with tissue paper which was moved circumferentially around the patient's upper and lower arm. Each finger was examined separately. The patients compared the sensation in the affected arm with the sensation in the unaffected arm [1]. Sensory testing of the upper limb was rated as normal or abnormal (heightened or diminished sensation). Upon detection of abnormal sensation, the investigators mapped the area and classified the findings as dermatomal or non-dermatomal using a dermatome and sensory innervation chart of the upper limb [30].

Reflexes of the biceps (C5-6) and triceps (C7-8) were tested bilaterally using a standard reflex hammer [31]. Each reflex was graded as reduced/absent, normal or increased compared to the unaffected side.

#### Upper limb neurodynamic tests

**Figure 1:**
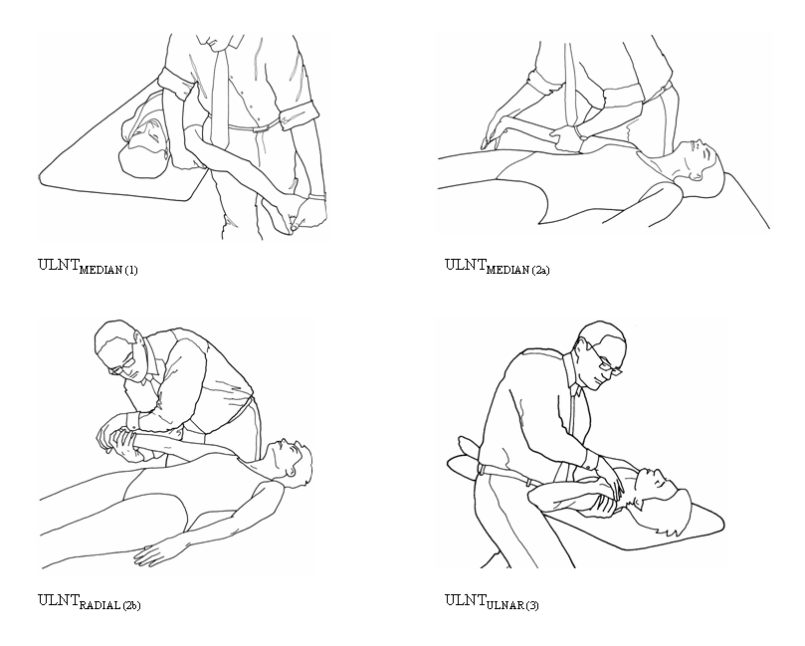
**End positions of upper limb neurodynamic tests**. Reprinted from Butler DS, The Sensitive Nervous System, Unley, DC, Australia: NOIgroup Publications, 2000, with kind permission.

ULNTs for the median nerve (ULNT_MEDIAN(1) _and ULNT _MEDIAN(2a)_), radial (ULNT_RADIAL(2b)_) and ulnar nerve (ULNT_ULNAR(3)_) were performed according to the operational definition described by Butler [25] (see Figure 1). The patient was positioned supine without a pillow. The hand of the untested side rested on the participant's abdomen. All ULNTs were performed with a standardised sequence (see Table 2). Movements were performed to the end of range or until symptoms were produced.

**Table 2:** ULNT sequencing

	1.	2.	3.	4.	5.	6.
ULNT_MEDIAN(1)_	Shoulder girdle fixation	Shoulder abduction	Wrist extension	Supination	Shoulder external rotation	Elbow extension
ULNT_MEDIAN(2a)_	Shoulder girdle depression	Elbow extension	Shoulder external rotation	Supination	Wrist extension	Shoulder abduction
ULNT_RADIAL(2b)_	Shoulder girdle depression	Elbow extension	Shoulder internal rotation	Pronation	Wrist flexion	Shoulder abduction
ULNT_ULNAR(3)_	Wrist extension	Pronation	Elbow flexion	Shoulder external rotation	Shoulder girdle depression	Shoulder abduction

Prior to performing the tests, the patients were instructed to communicate the onset of any sensation such as stretch, tingling or pain anywhere in the arm or neck. Once such a sensation was provoked, structural differentiation between neurogenic and non-neurogenic sources of pain was performed by the addition of sensitising movements at a site distant to the pain. The therapist could choose from the following sensitising movements: ipsi- and contralateral cervical lateral flexion, wrist extension or wrist flexion, or shoulder girdle elevation. If the patient's response was unclear, more than one of these sensitising movements were used.

Every test was performed on the unaffected arm first. These findings were then used as a reference for the affected side. An ULNT was considered positive if it reproduced the patient's symptoms at least partially and if structural differentiation supported a neurogenic source. The order of the four ULNTs was randomly assigned using randomisation software [32].

#### Nerve palpation

**Figure 2:**
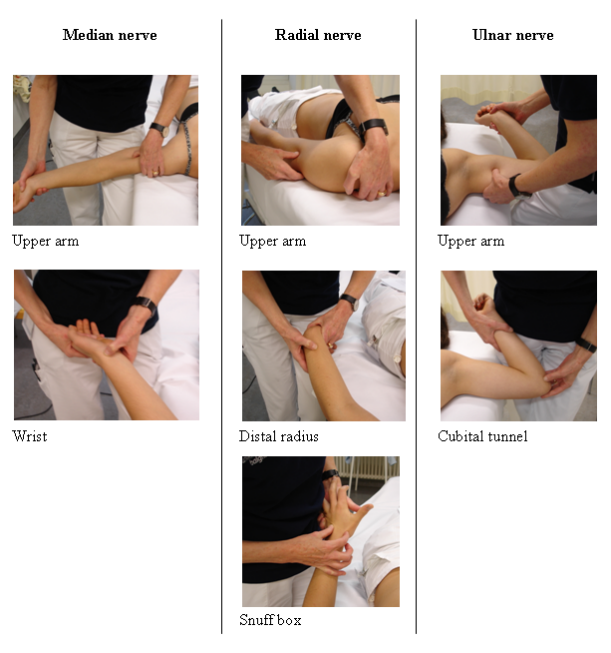
Peripheral nerve palpation points.

At least one proximal and one distal site along the radial, ulnar and median nerve were palpated with light to moderate pressure in random order. The palpated sites were chosen in regards to the accessibility of the nerve and involved the median nerve in the upper arm and the wrist, the radial nerve in the upper arm, at the distal radius and in the anatomical snuff box and the ulnar nerve in the upper arm and in the ulnar groove at the elbow (see Figure 2). Palpation was rated positive if pain or symptoms were elicited that were different to the unaffected side.

### Procedure

Bedside neurological examination, ULNTs and palpation were conducted by two experienced physiotherapists specialised in musculoskeletal therapy who were blinded to the patients' diagnoses. The investigators underwent 2 hours of training prior to the experiment in order to be familiar with the test performance and rating criteria. Both investigators examined each participant for 30 minutes. There was a break of 60 minutes between the two testing sessions to minimise a testing bias of the first on the second examination. The order of the two therapists was randomly allocated using a randomisation software [32]. Prior to testing, each investigator recorded the participant's current pain intensity on a visual analogue scale (VAS: 0–100) to verify whether pain intensity was comparable at the start of the two testing sessions.

The order of the bedside neurological examination, ULNTs and nerve palpation within each patient was the same for both testing sessions to ensure that a potential order effect was similar between testers.

We were interested in the reliability of the tests in isolation, i.e., irrespective from findings of a thorough patient interview or other clinical tests. Therefore, a separate clinician (rheumatologist) performed a brief patient interview and screened for any exclusion criteria. He then gave the patients concise explanations on the test procedure and familiarised them with neurodynamic tests by performing a straight leg raise with the addition of ankle dorsiflexion. Additionally, he determined joint range of motion of the upper limb and neck. The two investigators received information on symptom location and maximal joint range of motion. Joint range of motion is normally assessed before ULNT performance to identify possible joint stiffness which could affect the available range of motion during ULNTs.

### Statistical analysis

Kappa coefficients and standard errors [33] were calculated for each clinical test. Only test results of the affected side were used to avoid artificially inflated kappa values. Kappas were only computed for tests that had sufficient variability in their results [33]. The classification system proposed by Landis and Koch [34] was used to determine the level of reliability (poor: kappa smaller than zero; slight: zero to 0.20; fair: 0.21 to 0.40; moderate: 0.41 to 0.60; substantial: 0.61 to 0.80; almost perfect: 0.81 to 1.00).

A meta-analysis was performed to obtain an overall kappa value for those domains with multiple kappa values (MMT, ULNTs and palpation) by pooling the kappa values using a fixed effects model.

The difference between the kappas of the various domains was tested using a t-test with the level of significance set at 5% and ignoring the dependence of the samples. Statistical analysis was performed using Stata version 9.2 (4905 Lakeway Drive, College Station, USA).

## Results

The mean pain intensity at the start of the first session was 17.2/100 and 16.7/100 for the second session. There was no difference in the two VAS scores (paired t-test, p = 0.78), which demonstrates that the pain level at the start of each examination session was similar.

The frequency of positive ratings for each test is presented in Table 3. Sensory testing was positive in 42% of the patients (13% dermatomal; 29% non-dermatomal distribution). The frequency for a positive MMT for a specific level varied from 0–19%. The frequency for a positive ULNT was 31–39% depending on the variant and 13%–26% for the different palpation sites. No patient had abnormal biceps or triceps reflexes or positive MMT for C4.

**Table 3:** Frequency of positive findings and the reliability of clinical examination items

**Test**	**Frequency**		**Kappa** **(Standard error)**	**Test**	**Frequency**		**Kappa** **(Standard error)**
MMT				PALPATION			
C4	0/31	0%	NA	Median, upper arm	7/31	22.6%	0.50 (0.18)
C5	4/31	12.9%	0.53 (0.18)	Median, wrist	6/31	19.4%	0.79 (0.18)
C6	6/31	19.4%	0.90 (0.18)	Radial, upper arm	8/31	25.8%	0.66 (0.18)
C7	2/31	6.5%	0.45 (0.15)	Radial, distal radius	5/31	16.1%	0.67 (0.18)
C8	2/31	6.5%	0.64 (0.17)	Radial, snuff box	4/31	12.9%	0.61 (0.18)
T1	2/31	6.5%	1.00 (0.18)	Ulnar, upper arm	4/31	12.9%	0.36 (0.16)
				Ulnar, cubital fossa	5/31	16.1%	0.59 (0.18)
SENSORY TESTING							
Dermatomal Non-dermatomal	4/31 9/31	12.9% 29.0%	0.53 (0.13)				
							
						**Meta-analysis**	**Kappa (95% CI)**
						
ULNT
ULNT_MEDIAN(1)_	11/31	35.5%	0.54 (0.18)			MMT	0.68 (0.53, 0.83)
ULNT_MEDIAN(2a)_	11/31	35.5%	0.46 (0.18)			ULNT	0.45 (0.27, 0.63)
ULNT_RADIAL(2b)_	10/31	31.3%	0.44 (0.18)			Palpation	0.59 (0.46, 0.72)
ULNT_ULNAR(3)_	12/31	38.7%	0.36 (0.18)				

ULNT: Upper limb neurodynamic test, MMT: Manual muscle test, CI: confidence interval

Most tests showed moderate to substantial kappa values (see Table 3). Only palpation of the ulnar nerve in the upper arm and ULNT_ULNAR(3) _revealed fair agreement. MMTs for T1 and C6 demonstrated excellent intertester reliability. Since there were no abnormal findings for reflexes and MMT for C4, no reliability coefficient was computed for these variables.

The meta-analysis revealed a substantial intertester reliability for MMTs (overall kappa: 0.68; 95%CI: 0.53 – 0.83), a moderate reliability for ULNT (overall kappa 0.45; 95%CI: 0.27 – 0.63) and a moderate reliability for nerve palpation (overall kappa: 0.59; 95%CI: 0.46 – 0.72). Comparison of these results revealed no statistically significant difference in intertester reliability between test domains (MMT vs. ULNT p = 0.06, MMT vs. palpation p = 0.29, sensory testing vs. ULNT p = 0.35, sensory testing vs. palpation p = 0.37).

## Discussion

The findings of this study demonstrate that clinical tests to assess peripheral nerve injuries have moderate to substantial reliability when performed in patients who are referred with various neuromusculoskeletal conditions. Clinical tests for afferent and efferent nerve function showed moderate to substantial reliability and tests for increased mechanosensitivity (palpation and ULNTs) demonstrated moderate reliability. When compared statistically, there was no difference in the level of reliability for clinical tests for afferent/efferent nerve function and mechanosensitivity. The results of this study demonstrate that these clinical tests have a satisfactory level of reliability.

With respect to the reliability of ULNTs, most studies investigated whether symptoms occur at a consistent point in range [16-23]. These studies indicated that there is no difference in range of motion when different testers performed the tests. When appropriate reliability coefficients were reported, good to excellent values were observed for ULNTs both in a clinical and laboratory setting [20-23]. Other studies used symptom reproduction as the positive test criterion [35,36]. These studies showed only fair intertester reliability (kappa: 0.35 – 0.38). This discrepancy in reliability might not only be explained by the use of a different criterion, but the authors also suggested that poor test standardisation may have affected the outcome [35,36]. In addition, the ULNT_MEDIAN(1) _performed in the study by Vikarii-Juntura followed the earliest test description, which included elbow flexion rather than extension to load the median nerve [36]. This difference in test performance may also have had an impact on reliability.

Wainner et al. [26,37] reported substantial to almost perfect reliability for the interpretation of the ULNT_MEDIAN(1) _and ULNT_RADIAL(2b) _(kappa = 0.76 and 0.83, respectively). Although these authors used structural differentiation as one of three test criteria, a test could be rated as positive when the patients' symptoms were provoked with the test or when differences in range of motion were detected regardless of the outcome of structural differentiation. Hence, structural differentiation in line with a neurogenic source was not an essential criterion for the test to be considered positive.

In our study, structural differentiation was specifically included as an essential criterion to interpret ULNT outcomes. This may have introduced another source of variation, which may explain the somewhat lower reliability in our study compared to the findings by Wainner et al. [26]. However, structural differentiation is important to limit the amount of false positive results [38,39] and should in our opinion be included when interpreting ULNTs. Another difference between the two studies is the patient sample. Wainner et al. [26,37] only included patients referred to electrophysiological examination with suspected carpal tunnel syndrome or cervical radiculopathy. In the present study, we included patients referred with varying neuromusculoskeletal diagnoses. Although the two samples are markedly different, both are representative for patients in whom ULNTs are performed.

The reliability of individual nerve palpation tests was moderate to substantial. Interestingly, for both the median and ulnar nerve, there was a trend that palpation at distal sites was more reliable than palpation at proximal sites. However, this was not the case for the radial nerve. The level of reliability for nerve palpation observed in this study is similar to the results reported by Jepsen et al. [40] who also demonstrated moderate to substantial intertester reliability (kappa: 0.47 – 0.69).

Although bedside neurological examination is widely used by health professionals, its intertester reliability has been investigated scarcely in the type of patients commonly seen in a musculoskeletal clinic. Most studies demonstrated only slight to moderate reliability [26,36,40-43]. Our findings for sensory testing are in accordance with previous results [36]. However, our moderate to substantial findings for MMTs are higher than previously reported [26,36,43]. Jepsen et al. [43] used a 6 level scale to rate MMTs and Viikari-Juntura [36] a 3 level scale. We only used 2 levels in rating MMTs which may account for the higher reliability found in our study. However, Wainner et al. [26] reported lower reliability for MMTs using a similar 2-level scale. We assume that Wainner's population, which included a high percentage of patients with electrodiagnostically proven carpal tunnel syndrome and mild cervical radiculopathy, had a higher incidence of muscle weakness than our population. The fact that sensitivity of MMTs has been shown to be lower with small strength deficits [44] may explain the lower reliability in the study by Wainner et al. [26].

Several factors should be considered when interpreting the level of reliability found in this study. First of all, we did not use pressure algometry to measure and standardise palpation pressure. Manual palpation was chosen to closely replicate clinical practice. The reliability of palpation might be further increased if palpation pressure is quantified. Secondly, the examiners' decision may have been influenced by the outcome of preceding tests. It was however not practical to design the study in a way that the investigators were blinded from previous test outcomes. Thirdly, the fact that the two investigators were experienced physiotherapists with a specialisation in musculoskeletal therapy should be considered before generalising the results of this study. The investigators also received 2 hours training before conducting the testing procedures. Future research is required to investigate whether satisfactory levels of reliability can also be achieved in more novice clinicians without specific training. Finally, having demonstrated the reliability of these tests in isolation, a logical next step would be to investigate the reliability of the overall decision whether neuropathic mechanisms are present and whether subsequent interventions should target these mechanisms. Such an overall decision should be based on the patient interview and a series of clinical tests which further strengthen or weaken the hypothesis of nervous system involvement.

Reliability and validity are both essential clinometric properties of a test. This study focused on reliability, but there is increasing evidence that ULNTs have diagnostic merit [23,26,38,45,46]. There is however remarkably little literature on the validity of the bedside neurological examination and nerve palpation in patients with neuromusculoskeletal conditions. Future studies should concentrate on the further validation of clinical examination procedures for nerve function and mechanosensitivity.

## Conclusion

Clinical tests to evaluate increased nerve mechanosensitivity and afferent/efferent function have moderate to substantial reliability. This satisfactory level of reliability in combination with the increasing evidence of diagnostic merit [23,26,38,45,46] indicates that ULNTs have acceptable clinometric properties. Bearing in mind the different underlying pathophysiological mechanisms, clinicians should consider testing for both nerve function and nerve mechanosensitivity when diagnosing patients with suspected peripheral nerve lesions. However, the literature on the validity of bedside neurological examination in patients with musculoskeletal symptoms is scarce and there are no studies available which examine the validity of nerve palpation.

## Competing interests

The authors declare that they have no competing interests.

## Authors' contributions

AB designed the study, collected data and prepared the manuscript. FB and SK assisted in the design and data collection. HL and MC were involved in the design and drafting of the manuscript. UH and LB performed the statistical analysis and were involved in drafting the manuscript. All authors read and approved the final manuscript.

## Pre-publication history

The pre-publication history for this paper can be accessed here:


